# Exploration of Environmental DNA (eDNA) to Detect Kirtland’s Snake (*Clonophis kirtlandii*)

**DOI:** 10.3390/ani10061057

**Published:** 2020-06-19

**Authors:** Rikki Ratsch, Bruce A. Kingsbury, Mark A. Jordan

**Affiliations:** Department of Biology, Purdue University Fort Wayne, Fort Wayne, IN 46805, USA; rrats063@outlook.com (R.R.); kingsbur@pfw.edu (B.A.K.)

**Keywords:** conservation, coverboard survey, DNA degradation, crayfish burrow, qPCR, habitat preference

## Abstract

**Simple Summary:**

Small and difficult to find species of conservation concern such as the Kirtland’s Snake require significant survey effort using traditional methods. Surveying for DNA shed into the environment, or environmental DNA, we set out to improve detection probability and efficiency to aid in future conservation efforts for this species. Field surveys revealed temporal and spatial variation in Kirtland’s Snake activity. More snakes were found in the spring, during the first field season, and in areas with abundant grass, herbaceous vegetation, and shrubs. We collected environmental samples and developed a molecular assay to detect eDNA across this spatial and temporal gradient of snake activity. We also tested the persistence of DNA in the microenvironment snakes are expected to use by introducing feces into artificial burrows. We were able to detect snake eDNA in only a single environmental sample and found that eDNA in artificial burrows appears to decline within a week. We explored the potential methodological and biological causes of this low detection success to aid future research employing eDNA detection as a survey method in snakes.

**Abstract:**

Environmental DNA (eDNA) surveys utilize DNA shed by organisms into their environment in order to detect their presence. This technique has proven effective in many systems for detecting rare or cryptic species that require high survey effort. One potential candidate for eDNA surveying is Kirtland’s Snake (*Clonophis kirtlandii*), a small natricine endemic to the midwestern USA and threatened throughout its range. Due to its cryptic and fossorial lifestyle, it is also a notoriously difficult snake to survey, which has limited efforts to understand its ecology. Our goal was to utilize eDNA surveys for this species to increase detection probability and improve survey efficiency to assist future conservation efforts. We conducted coverboard surveys and habitat analyses to determine the spatial and temporal activity of snakes, and used this information to collect environmental samples in areas of high and low snake activity. In addition, we spiked artificial crayfish burrows with Kirtland’s Snake feces to assess the persistence of eDNA under semi-natural conditions. A quantitative PCR (qPCR) assay using a hydrolysis probe was developed to screen the environmental samples for Kirtland’s Snake eDNA that excluded closely related and co-occurring species. Our field surveys showed that snakes were found in the spring during the first of two seasons, and in areas with abundant grass, herbaceous vegetation, and shrubs. We found that eDNA declines within a week under field conditions in artificial crayfish burrows. In environmental samples of crayfish burrow water and sediment, soil, and open water, a single detection was found out of 380 samples. While there may be physicochemical and biological explanations for the low detection observed, characteristics of assay performance and sampling methodology may have also increased the potential for false negatives. We explored these outcomes in an effort to refine and advance the successful application of eDNA surveying in snakes and groundwater microhabitats.

## 1. Introduction

Environmental DNA (eDNA) assays represent a survey technique that has seen increasing attention and use in wildlife management. The method involves taking environmental samples such as water or soil and testing the material for the presence of short strands of DNA that were shed by organisms into the environment [1]. Over the last few years, eDNA analysis has been often used for the detection of low numbers of individuals at the leading edge of invasive species. Some of these include American Bullfrogs (*Lithobates catesbeianus*) in France [2,3], Asian Carp (*Hypophthalmichthys* spp.) in the midwestern USA [4,5,6], and Burmese Pythons (*Python bivittatus*) in Florida [7,8]. Threatened and endangered species also present an opportunity to apply eDNA analysis as their rarity and legal protection can impede standard survey methods [9]. Listed as threatened or endangered in most areas they occur, the Eastern Hellbender (*Cryptobranchus alleganiensis*) is of high conservation concern and eDNA monitoring has already been used to detect their presence in streams [10,11]. Similarly, eDNA analysis has already been used for detection of the threatened Great Crested Newt (*Triturus cristatus*) in the UK [12]. Other threatened species that have been successfully detected with eDNA analysis include the Eastern Massasauga (*Sistrurus catenatus*) and the Bull Trout (*Salvelinus confluentus*) [2,13,14,15].

The ability to detect eDNA depends on its rate of production and its persistence in the environment [16]. Production rates have been shown to vary with the abundance or biomass [17,18,19] and seasonal activity [11,20,21]. It also appears to vary among taxa in relation to the intrinsic rate that cells are shed from individuals [22]. Once produced, the persistence or degradation rate is critical for detection of DNA in the environment. A short lifespan is ideal for immediate presence information; however, if the DNA degrades too quickly, it will reduce overall ability to detect species. A longer DNA lifespan allows for greater chances for detection, but at the cost of accuracy relating to the current presence or absence of a target species. Many environmental factors can influence the persistence of eDNA, including microbial activity, pH, temperature, conductivity, substrate type, and UV radiation [23,24,25,26]. Sampling in a way that minimizes these influences will increase the longevity of eDNA, thus potentially increasing the probability of detection. Previous studies have shown a wide variation of eDNA persistence in the water column, ranging from less than one day to nearly 60 days [8,9,19,26,27,28,29]. Conversely, eDNA present in sediment has been shown to remain intact for months [30,31].

Kirtland’s Snake (*C. kirtlandii*) is small and poorly understood due to its secretive, fossorial nature [32,33,34]. They are most often found in open areas that are wet or prone to seasonal flooding, under cover, buried in leaf litter, or inside burrows. Their preferred habitats are moisture-rich open meadows or prairies, but they can also be found in swamps, bogs, and forests containing pools or creeks [35,36]. However, the most well-known populations of *C. kirtlandii* occur in urban environments with grassy areas containing ample cover, a nearby water source, and crayfish burrows [37,38]. Regardless of habitat, the presence of *C. kirtlandii* is strongly associated with nearby water bodies and crayfish burrows, the latter of which they use as refugia, hibernacula, and to seek prey or moisture [35,39,40]. Seasonally, observations of *C. kirtlandii* peak during spring, followed by a drop off into summer before increasing once more in the fall [41]. It is believed that the snakes become nocturnal, retreat below ground to areas of higher moisture, or potentially go into aestivation during summer months [32,34,36].

*Clonophis kirtlandii* is endemic to the midwestern USA with populations currently residing in the states of Michigan, Illinois, Indiana, Ohio, and Kentucky [35,40]. Their populations are patchy and reduced compared to historic records [35,42]. They are listed as state endangered in Michigan, Indiana, and Kentucky as well as having a state-threatened listing in Illinois and Ohio. Due to their cryptic and fossorial nature, it is difficult to assess the status of their populations. Most available information is restricted to simple presence or absence data. Traditional survey methods are difficult and expensive to conduct for this species, and the general lack of ecological knowledge restricts the ability to make informed management decisions [41]. This is underlined by a recent federal status assessment for *C. kirtlandii*, in which the species was unable to be listed as threatened or endangered due to the lack in overall understanding of both the health of their populations and the potential threats to them [42].

Previous success in the application of eDNA to detect rare and cryptic species across various settings led us to develop and evaluate the effectiveness of an eDNA assay to survey for *C. kirtlandii*. In particular, we surmised that their use of crayfish burrows would be advantageous for environmental sampling given that burrows are relatively contained aquatic microhabitats where eDNA may persist under cool, dark conditions. We addressed three primary objectives: (1) the development of an environmental DNA assay that is specific to *C. kirtlandii* that excludes other closely related and co-occurring snakes; (2) the determination of the detectability and degradation rate of *C. kirtlandii* eDNA in crayfish burrows; and (3) the comparison of eDNA and coverboard surveys to detect *C. kirtlandii*.

## 2. Materials and Methods

### 2.1. Snake Surveys

#### 2.1.1. Field Site

The field location for this study was Muscatatuck National Wildlife Refuge (MNWR) in Jackson and Jennings counties, Indiana (38° 55′ 58” N, 85° 48′ 32” W). Approximately half of the refuge’s 7850 total acres are comprised of bottomland hardwood forest [43]. Upland hardwood forest, agriculture areas, and wet herbaceous areas comprise another 40% of the refuge. The remaining 10% of the refuge land is composed of upland herbaceous plants, plantations, shrubs, and developed areas. About 2700 acres of MNWR is flooded on an annual basis.

#### 2.1.2. Coverboard Surveys

Records of *C. kirtlandii* densities from previous surveys conducted from 2008 to 2010 were used to select study sites within MNWR (Evin Carter, pers. comm.). Our surveys utilized artificial coverboards placed at 30-m intervals alongside bodies of water to capitalize on the species’ strong association to groundwater. The coverboards were placed into 11 transects split into three groups, north (n = 4), middle (n = 4), and south (n = 3). Transects varied in length from 7 to 36 boards, and in total 193 coverboards were used. All boards were surveyed and replaced as needed on a weekly basis from May into October 2017 and April into July 2018.

Coverboards consisted of a 0.95-cm thickness Saturn brand under carpet foam padding with an attached plastic moisture barrier to retain water absorbed by the foam (Vicky Meretsky, pers. comm.). This created a warm and moist microhabitat ideal for *C. kirtlandii.* Coverboards were cut into 30 cm × 60 cm rectangles for easy handling and held to the ground with two 10.80 cm × 3.18 cm yard staples to prevent them from being washed away by flooding. At the start of each transect, time, temperature, and cloud cover weather were recorded. Temperature was recorded via alcohol thermometer and cloud cover percentage was visually estimated. Time was also recorded at the end of each transect to give survey duration. 

During the first season, a single ventral scale clip immediately anterior to the divided anal scale was taken from all snakes encountered, including *C. kirtlandii*. This enabled us to identify recaptures and collect tissue samples. During the second season, *C. kirtlandii* were individually marked by standard ventral scale clipping protocol and tail clips were also collected [44]. Clippings from marked snakes were placed in a sterile 5 mL tube filled with 95% ethanol and stored at −80 °C until needed for DNA extraction. Equipment was flame sterilized before and after each use.

#### 2.1.3. Feces Collection

At the start of each field season, a maximum of 10 *C. kirtlandii* were captured and held in captivity for up to two weeks to attain ejecta (feces and urates) for use as field positives and in the degradation experiment (see below). Snakes were housed individually in plastic tubs measuring 58 cm × 41 cm × 15 cm. Each tub contained a hide, a water dish, and a heat pad to allow for thermoregulation. Ejecta was collected using sterilized forceps and disposable pipettes and placed in sterile 10 mL tubes containing 5 mL of distilled water to improve sample handling. The samples were weighed with a digital balance and stored in a −80 °C freezer until needed. Once sufficient ejecta had been collected, or after a snake had been held for two weeks, snakes were returned and released at their capture site.

#### 2.1.4. Habitat Data

A simple set of habitat variables was collected at coverboards during June of the second field season to see if any had a significant influence on the presence of *C. kirtlandii*. These variables included canopy cover, ground cover, dominant vegetation, and distance to a set of landmarks. Canopy cover was recorded using a densiometer and following standard protocols to average total coverage from all cardinal directions [45]. The distances to logs with a diameter greater than 10 cm, trees greater than 5 cm diameter at breast height (DBH), and shrubs were measured up to a distance of 30 m. Ground cover categories included water, rock, bare ground, leaf litter, grass, herbaceous vegetation, and shrub. Using a modified line transect method, ground cover percentage was estimated at coverboards by extending a meter tape 3 m in each cardinal direction and recording the ground cover in a 30 cm path [46,47,48]. Similar to canopy cover, the total ground cover percentages were averaged between all cardinal directions. To reduce effort and retain enough samples for statistical analysis, habitat data were only collected at even-numbered coverboards. This resulted in data being collected from 96 coverboards instead of all 193. 

### 2.2. Environmental DNA 

#### 2.2.1. Assay Design

To design a species-specific assay for *C. kirtlandii*, the cytochrome oxidase subunit I (COI) gene of the mitochondrial genome was selected to exclude other closely related and co-occurring natricine snakes. COI has been shown to be highly variable between species, but far less so within, allowing for specificity between closely related co-occurring species [49,50,51]. National Center for Biotechnology Information (NCBI) Primer-BLAST (https://www.ncbi.nlm.nih.gov/tools/primer-blast) and the Integrated DNA Technologies (IDT) PrimerQuest Tool (https://www.idtdna.com/Primerquest) were used to generate 10 potential primer sets as well as their complimentary probes closely following the NCBI and IDT guidelines. In silico testing was conducted using snake COI sequences from GenBank (https://www.ncbi.nlm.nih.gov) aligned in Geneious (https://www.geneious.com) to look for base pair mismatches. For a primer pair to be considered for further testing, a collective mismatch of at least four base pairs was required between *C. kirtlandii* (GenBank KU986171.1) and non-target species that: (1) co-occur in the counties of the study site [38]; or (2) have a close phylogenetic relationship [52] with an overlapping geographic range. Those species included: Dekay’s Brownsnake (*Storeria dekayi*), Red-belled Snake (*S. occipitomaculata*), Common Watersnake (*Nerodia sipedon*), Plain-bellied Watersnake (*N. erythrogaster*), Gray Ratsnake (*Pantherophis spiloides*), Eastern Milksnake (*Lampropeltis triangulum*), Queensnake (*Regina septemvittata*), Smooth Earthsnake (*Virginia valeria*), Eastern Ribbonsnake (*Thamnophis sauritus*), Western Ribbonsnake (*T. proximus*), Plains Gartersnake (*T. radix*), Common Gartnersnake (*T. sirtalis*), and Butler’s Gartersnake (*T. butleri*) (Table S1). Candidate primer pairs were then tested for specificity against the full NCBI nucleotide database in Primer-Blast. We maximized the potential for matching an unintended sequence in the database by requiring a single mismatch within 5 bp of the 3′ end of either primer [53].

Candidate primers and probes were also evaluated in the IDT OligoAnalyzer Tool (https://www.idtdna.com/calc/analyzer) to analyze their melting temperature (T_m_), guanine and cytosine content (percent GC), primer hairpin (**Δ**G), and primer dimerization (**Δ**G). Primers designed were 15–25 base pairs (bp), would create a 70–200 bp amplicon, had a GC content of 40–60%, and had a T_m_ of 57–63 °C with a maximum of 2 °C difference between primer pairs. They were also double-checked to assure at least one mismatch between them and non-target species to limit cross amplification. Other selection parameters for primers were a **Δ**G (kcal/mol) more positive than −9.0 to limit hairpin formation and dimerization. Once a primer set was established, a complimentary probe was generated in PrimerQuest and evaluated by OligoAnalyzer for the same parameters as the primers. However, the probe parameters were slightly modified, with 20–30 bp considered, the T_m_ 6–8 °C higher than the primers, GC content of 35–65%, annealing temperature (T_a_) less than 5 °C below the primers, and a **Δ**G (kcal/mol) more positive than −9.0. The probe was also not to overlap with either the forward or reverse primers. The primers and probe were ordered as DNA oligonucleotides from IDT and the probe utilized a FAM fluorescent dye and ZEN quencher. 

In vitro testing was conducted with tissue derived DNA extracts (0.1 ng/µL) from both *C. kirtlandii* as well as commonly co-occurring natricine snakes including *S. dekayi*, *S. occipitomaculata*, *N. sipedon*, *T. sirtalis*, and *T. sauritus*. A range of annealing temperatures was tested to optimize amplification (2 °C increments from 42 to 50 °C, 55 °C, and 1 °C increments from 60 to 65 °C) using 0.3 µM primer and 0.2 µM probe concentrations. Primer and probe concentrations were also varied to optimize the assay (at T_a_ = 60 °C, primers = 0.5 µM and probe = 0.2 µM; and at T_a_ = 62 °C, primers = 0.15 or 0.3 µM and probe = 0.1 µM). Assay performance was tested with known copy number DNA standards included on most qPCR plates to allow for the assessment of assay efficiency and quantification of starting copy number in unknown eDNA samples. DNA standards (further described in Section 2.2.5) consisted of a synthetic dsDNA oligo (gBlock) from IDT diluted in a series of known DNA copy numbers/µL (10^1^–10^6^). This allowed for the assessment of assay efficiency and quantification of starting copy number in an eDNA sample. PCR products from a positive field sample, a tissue extraction, and the known DNA template (gBlock) were Sanger sequenced by GENEWIZ (https://www.genewiz.com) to confirm assay efficacy and specificity. 

#### 2.2.2. Environmental DNA Sample Collection 

To understand eDNA detectability across microhabitats used by *C. kirtlandii*, we collected five types of environmental samples at each point sampled: water, sediment, and soil from crayfish burrows, soil from under artificial cover objects, and water from adjacent open water. It is important to distinguish that burrow water and burrow sediment were collected as a single field sample and separated once in the lab. All other field sample types were collected and processed individually. To better distribute the limited sample points, samples were collected from every other cover object (60-m spacing) at two transects chosen based on a contrast in snake activity from preliminary surveys. To test the effectiveness of our protocol, the two eDNA sample transects included one of relative high snake activity (S3) and the other with low snake activity (M1). These two sample locations were approximately 3 km apart so that *C. kirtlandii* migration between them within a season was unlikely. In a sampling event, 10 samples of each of the five environmental types were collected at the S3 and M1 locations resulting in 100 samples. In 2017, three sampling events (May 30, July 26, and October 25) were conducted to assess seasonal variation in eDNA presence giving a total of 300 samples for the year. We repeated this protocol in spring 2018 (May 23) but collected only crayfish burrow water and sediment at each location resulting in 40 samples. Due to low snake captures in the 2018 season, a parallel set of crayfish burrow samples was also taken spaced 30 m away from the coverboards giving 80 samples for the year. This was done to determine if *C. kirtlandii* were near to, but not utilizing the coverboards due to a reduction in the water table that year.

To draw water and sediment from crayfish burrows, a 60-mL syringe with 1m length of 3.175-mm diameter PVC tubing was used [54]. The tube was guided as far as possible down the burrow and used to draw a 50-mL sample of water and sediment. The sample was then placed into a sterile 50-mL centrifuge tube. Due to the limited amount of water and high levels of sediment in crayfish burrows, volumes greater than 50 mL could not be reliably collected and processed. At the same borrows as the water and sediment samples, a 5-g sample of soil was collected from the lip of the burrow using a disposable spatula and placed into a separate 50-mL tube. A 5-g sample of soil was also scooped at random from under artificial cover objects and placed into a 50-mL tube. Finally, 500 mL of water was sampled from an adjacent open surface water source by immersing a sterile 500-mL polypropylene sample bottle into the water until full. This volume was selected as a balance among sample volume, sample number, storage space, and clogging of filtration units. After collection, all samples were placed into a cooler filled with ice for transportation back to the laboratory where they would be refrigerated and filtered within 24 h (see Section 2.2.4). Negative and positive field control samples were included with each day of sampling effort. The negative field control consisted of a closed 50-mL centrifuge tube of autoclaved reverse osmosis water brought into the field and stored alongside all other samples in the field cooler [4,10]. The positive field control was created by placing collected feces from one *C. kirtlandii* into a crayfish burrow water and sediment sample. 

#### 2.2.3. Environmental DNA Degradation Study

Degradation of *C. kirtlandii* eDNA was conducted in situ using collected snake feces to spike artificial crayfish burrows. A coverboard transect with no *C. kirtlandii* captures was selected as a degradation study site (M4). Following the coverboard transect along the edge of the water, 10 artificial crayfish burrows were placed at 10-m intervals. To create the artificial burrows, 3.81-cm diameter PVC piping was cut to lengths of 76.2 cm and then a post-hole digger was used to bury those pipes in the ground. Opposing pairs of 3-mm holes were drilled into the pipes at 8-cm intervals and the bottom of the pipe was left open to allow for water to fill the pipe. Vents were added to the top of the pipes to keep animals from entering and becoming trapped, but still allow for rainwater to enter. After a settling period of two weeks, a sample of *C. kirtlandii* feces was added to each of the pipes. To mimic natural variation in feces weight, each pipe was spiked with a single fecal sample ranging from 0.02 to 2.50 g. Nine sample sets were collected from each of the 10 pipes. They included a pre-spike sample and a post-spike sample 3 h after the addition of feces as well as subsequent samples collected at Days 1, 2, 3, 10, 17, 25, and 31 after spiking the artificial burrows. Sampling followed the same methods used with crayfish burrows to collect paired water and sediment samples. Temperature, pH, and dissolved oxygen in water collected from the pipe was recorded. Water level in the pipes varied throughout the experiment due to water table variation, rainfall, and removal of water for sampling. We measured the water level of each pipe at each sampling event and calculated water volume from pipe dimensions for use in analyses. The water volume data also allowed us to normalize the weight of remaining feces due to removal sampling.

#### 2.2.4. DNA Capture and Extraction 

DNA extraction from environmental samples utilized a modified CTAB (cetyltrimethylammonium bromide) and Sevag (chloroform:iso-amyl 24:1 alcohol) protocol developed by Coyne et al. and used by Turner et al. [6,31,55,56,57]. Five grams of 360,000 molecular weight polyvinyl-pyrrolidone (PVP) were added per 500 mL of CTAB to mitigate potential inhibition through plant-based polyphenols during qPCR [58]. Once in the lab, crayfish burrow environmental samples were centrifuged at 3000 RPM at 4 °C for 10 min to separate the suspended sediment from the water [54]. The supernatant was then passed through a 0.2- or 0.45-µm PES membrane (Nalgene^TM^ Rapid-Flow^TM^ Sterile Disposable Filter Units) for the 50- and 500-mL samples, respectively [59]. The 500-mL open water samples utilized the 0.45-µm membrane to minimize clogging issues at larger volumes [57,60]. A vacuum pump generating 10–35 PSI was used to pass the fluid through the membrane. Forceps were used to tear, fold, and place the membranes into a 2-mL microcentrifuge tube before adding enough CTAB buffer solution to completely cover them (1 mL). Between filters, forceps were immersed in 10% bleach, rinsed with distilled water, and flame sterilized. Centrifuged sediment pellets and soil samples were processed by the addition of 2 mL of CTAB buffer per gram of sediment [57] (up to a maximum of 15 mL). All samples remained immersed in CTAB for two weeks at room temperature with mild agitation every third day to maximize DNA recovery [58]. After two weeks, the full volume of CTAB was run through their respective sediment or filter extraction process [61] and placed into a −80 °C freezer for storage until needed for qPCR. A separate laboratory was dedicated to the eDNA capture and extraction procedures, and the workspace was sterilized with 10% bleach before and after laboratory work.

Negative controls of autoclaved reverse osmosis water were added to each extraction set to assess potential contamination occurrence and to distinguish between collection-based and extraction-based contamination. Crayfish burrow water and sediment samples were also passed through a Zymo DNA Clean & Concentrator-5 kit following the 5:1 binding buffer to sample ratio DNA fragment protocol and eluted in 15 µL of buffer. Tissue samples of *C. kirtlandii* and non-target species were included in qPCR to test for cross amplification of the eDNA assay (described below). These extractions were done with a Qiagen DNeasy Blood and Tissue Kit following the standard tissue extraction protocol. After extraction, they were all subsequently diluted to 0.1 ng/µL for use in PCR. All tissue-based DNA extractions were performed in a separate laboratory and DNA derived from tissues was not allowed in the eDNA workspace to minimize potential contamination. 

#### 2.2.5. Quantitative PCR Protocol

All 15-µL qPCR reactions were conducted on 96-well Thermoscientific AB-2800/W qPCR plates and contained 0.3 µM of each primer and 0.2 µM of the *C. kirtlandii* probe following optimization of conditions (primer and probe concentrations, Ta). A 1× concentration of reaction mix (Bio-Rad SsoAdvanced Universal Probes Supermix), Internal Positive Control master mix, and IPC DNA (Applied Biosystems TaqMan Exogenous Internal Positive Control) was also added. To this, 2 µL of DNA were added to reach the desired volume in each well. All reactions were done in triplicate to improve detection and negate the effects of well-to-well variation. A field sample was considered positive for *C. kirtlandii* if 2 of 3 technical replicates were positive; however, we also evaluated patterns when relaxing this threshold to 1 of 3 replicates. The reaction conditions started with a 95 °C denaturation for 10 min followed by 50 cycles of 95 °C for 15 s and 60 °C for 30 s. The reactions were conducted on a Bio-Rad CFX96 Real-Time System and FAM (probe) and VIC (IPC) fluorophores were used. Negative and positive controls were added to each plate to determine contamination and to check proper reaction function, respectively. The qPCR plates also contained the dilution series of DNA standards (as described in Section 2.2.2) in triplicate to evaluate reaction efficiency and R^2^ values. We used the lowest number of copies to give at least one positive as the limit of detection (LOD) and the lowest number of copies to be detected at all three PCR replicates as the limit of quantification (LOQ) [62].

### 2.3. Statistical Analyses 

Binary logistic regression was used to test what habitat variables had a significant impact on the presence of *C. kirtlandii*. Before the regression was applied, variables with low sample sizes were removed from further analysis (ground cover water, bare dirt, and rock). The remaining variables (canopy cover, distances to log, tree, shrub, and ground cover of leaf, grass, herbaceous, and shrub) were then placed into the regression using backward stepwise (Wald) removal. 

A general estimating equation (GEE) was used to evaluate the effect that transect group, date, time of day, temperature, and cloud cover had on the number of snakes captured. Transect was used as the repeated measures subject in the analysis. We used a negative binomial model due to large numbers of zeros in the dataset, which resulted in overdispersion (large variance compared to the mean count). An AR (1) correlation matrix was used with the model as variables such as temperature are related to time of day or seasonal variation, thus become less correlated over time. Transect group (n = 3) and year (n = 2) were used as factors. The south transect group and year 2018 were used as reference categories, thus were set to zero in the model. Day of year, survey start time, temperature, and cloud cover were used as covariates and total snake captures at a transect on a sample day was the dependent variable. 

For eDNA degradation testing, a GEE was used with a binary logistic model to test what variables had a significant impact on eDNA detection in the concentrated sediment samples. Before the model was run, a correlation matrix of independent variables was created to assess multicollinearity. Temperature, pH, and dissolved oxygen were removed from further analysis due to high correlation with water volume. Pipes were used as the repeated measures subject in the model (n = 10) while days, feces weight, and water volume were used as covariates. The dependent variable used was positive eDNA detection. An unstructured working correlation matrix was selected for this model to allow for correlation between repeated measures, but not constrain the relationships between the variables. All statistical analyses were performed on IBM’s Statistical Package for the Social Sciences (SPSS) [63]. 

As there were a handful of positive detection recorded from all the eDNA field samples, no statistical analyses were possible for these data.

## 3. Results

### 3.1. Coverboard Surveys

**Figure 1:**
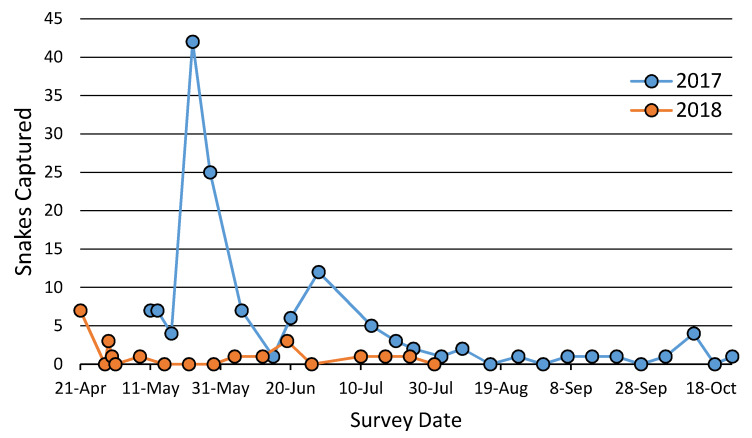
*Clonophis kirtlandii* captures over time from coverboard surveys. A peak in snake captures was observed on 23 May 2017. No peak in snake activity occurred in 2018.

**Figure 2:**
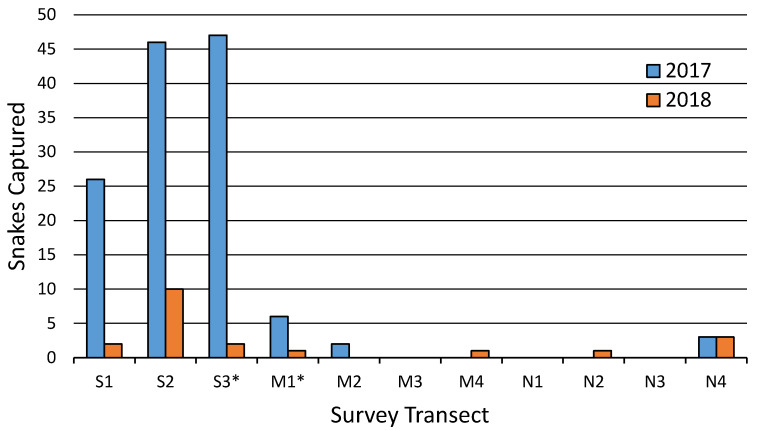
*Clonophis kirtlandii* captures between coverboard transects. More snakes were captured in 2017 and the majority of snakes were captured from the S transects. Asterisks mark transects used for eDNA sampling.

Captures of *C. kirtlandii* varied both temporally and spatially (Figure 1 and Figure 2). Transect group (*p* < 0.0001), year (*p* < 0.0001), and day of year (*p* < 0.0001) were related to the number of snakes captured in the GEE of coverboard survey variables (Table 1). The middle and north transect groups, in addition to day of year, had negative coefficients with snake captures while year 2017 had a positive coefficient with captures. 

In 2017, 25 surveys were conducted from May to October resulting in 134 captures. Of that total, 22 snakes (16.4%) were recaptures. In 2018, 17 surveys were conducted, resulting in 20 snakes captured with a single recapture (5%). Late spring was the most successful time of the year. The greatest number of snakes found in a single day was 42 on 23 May 2017. Due to low overall snake numbers in 2018, no peak was observed that year. 

Spatially, *C. kirtlandii* were relatively more abundant in the south group of transects compared to the middle and north transect groups (Figure 2). Between the two survey seasons, 134 snakes were captured in the three south transects while only 20 snakes were captured in the other eight transects. Within the south group, Transects 2 and 3 had the greatest overall number of snake captures at 57 and 49, respectively. 

Three habitat variables were removed due to high zero counts out of the 96 boards measured. These were ground cover percentage of water (n = 4), bare ground (n = 10), and rock (n = 6). In the final model, three habitat variables remained statistically significant, with a higher probability of snake presence with closer distance to shrub (*p* = 0.041), and higher ground cover of grass (*p* = 0.044) and herbaceous vegetation (*p* = 0.048) (Table 2). Distance to log and percent ground cover shrub were included in the final model, but were not statistically significant.

### 3.2. Assay Design

All primer pair candidates showed either high dimerization or cross amplification with non-target species. To find an effective primer pair, all forward and reverse primers were cross matched to design a new primer set. A 20-base forward (Clonophis_CO1_FP5: 5′-TCC CCT TGT TCG TTT GGT CA-3′) and a 19-base reverse (Clonophis_CO1_RP4: 5′-CAC CTC CGC ATG GAT CGA A-3′) primer were selected. This primer pair had the lowest dimerization, with the most negative **Δ**G at −7.13 kcal/mol, and showed the least cross amplification during gel visualization. When tested for specificity in Primer-Blast against the full nucleotide database in GenBank, no target other than *Clonophis* COI was recovered. It produces a 135-bp amplicon spanning 426–560 of *C. kirtlandii* COI gene. Within this sequence, a 26-base probe (Clonophis_CO1_PR: 5′-ACC GAC CGA AAC ATT AAC ACC TCC TT-3′) was selected and fit at the 3′ end without overlapping the reverse primer. Alignment of this primer and probe combination to CO1 with co-occurring or closely related natricine snakes showed a range of 5–9 nt base differences (Table S1). 

During in vitro testing with gel electrophoresis, the selected primer set cross-amplified with *S. dekayi*, *T. sauritus*, and *T. sirtalis*. Optimization of T_a_ and primer and probe concentrations did not fully resolve cross amplification, but non-target species amplified at a far later cycle than *C. kirtlandii* (Figure S1). With 0.1 ng/µL tissue samples, non-target species crossed the detection threshold at or after 40 cycles while *C. kirtlandii* was crossed at 24 cycles. To eliminate false positives in qPCR results, a cutoff point was set at cycle 40. Any amplification after this cycle was classified as spurious and considered a negative. Since eDNA concentrations are typically much lower than the concentration used in these tests, the assay was judged to have sufficient resolution to exclude non-target species for subsequent analysis of field collected environmental samples (Figure S2).

The average amplification efficiency of the assay was 80% and the assay had an R^2^ of 0.899. A standard curve equation of y = −3.9774x + 46.498 was created by plotting starting quantities of the DNA standards against the Cq values for all of the plates ran (Figure S3). The LOD was 100 and LOQ was 1000 starting copies/µL of *C. kirtlandii* DNA, respectively.

### 3.3. Environmental DNA Surveys

In total, 380 eDNA field samples were collected from transects S3 and M1 between the two field seasons. Out of all samples, sediment from one crayfish burrow in May 2017 at the S3 transect resulted in a detection when requiring two of three positive qPCR replicates. Three additional burrows were found to be positive when the threshold for detection was relaxed to one of three positive qPCR replicates. Two were in the S3 transect (one in May 2017 and one in 2018), and one was in M1 transect in 2018. Two open water samples in July 2017 had one amplification using one of three qPCR replicates, with none detected at the two of three threshold. No qPCR inhibition was found in crayfish burrow water or sediment, or in open water, but that was not the case with soil samples from under coverboards (51.7% inhibited) and crayfish burrow lips (8.3% inhibited). The absence of detections in uninhibited samples and available resources led us to not pursue soil samples further. Importantly, all field positives of spiked feces resulted in positive detections using the two of three threshold for qPCR replication in both water and sediment samples and no detections occurred in any field or lab negatives.

### 3.4. Environmental DNA Persistence

During the degradation study using artificial crayfish burrows, eight sample sets over time were collected, including one prior to spiking *C. kirtlandii* feces into the pipes. Due to natural water fluctuation, the Day 17 sample set was not able to be collected as the pipes completely dried out that week. One sample did not have a recorded initial weight, so the average initial weight of the other nine samples was substituted (0.72 g). This allowed for all 10 pipes to be used in the statistical analysis. No pre-spike samples had a positive detection. Among the 10 pipes, burrow sediment samples had seven or eight positive detections through Day 3, declining to two or fewer by Day 10 using the threshold of two of three positive qPCR replicates (Table 3). When using a threshold of one of three positive qPCR replicates, there were 7–9 positive detections through Day 3. On and after Day 10, there were one or two additional detections with this lower threshold. There were many fewer detections in burrow water under both thresholds.

**Figure 3:**
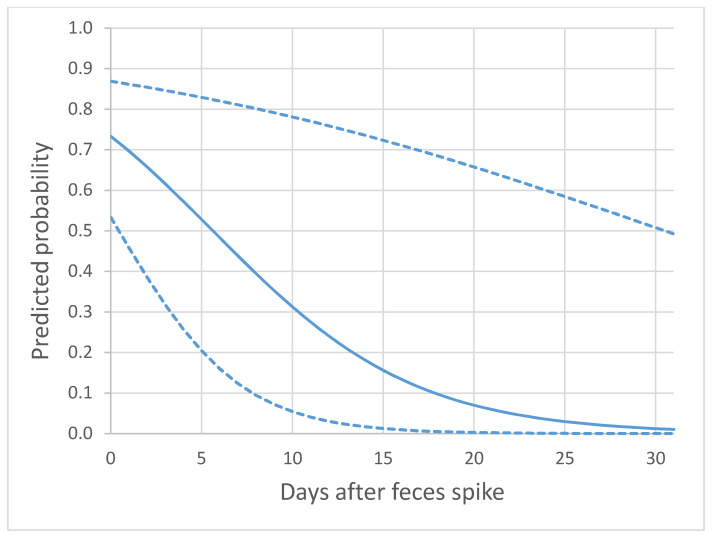
Predicted probability (mean (solid line) and 95% confidence interval (dashed line)) of detecting *C. kirtlandii* eDNA over time from the sediment of experimentally spiked artificial crayfish burrows. The binary logistic regression model was derived from the general estimating equation analysis, using days following spike as the single predictor of eDNA detection when two of three qPCR replicates was used as the threshold for detection (intercept = 1.012, β = −0.180).

We retained water volume as potential predictor of detection in artificial burrows after finding correlations between water volume, temperature, dissolved oxygen, and pH (Table S2). To evaluate predictors of eDNA persistence, we used presence data based on two of three qPCR replicates. Days post-spike was found to predict eDNA presence (β = −0.164, *p* < 0.0001) in the GEE while water volume (*p* = 0.24) and feces weight (*p* = 0.77) did not. Decline in the probability of detection of eDNA was found after spiking artificial burrows (Figure 3). The mean probability of detection of eDNA was estimated to be 0.73 on Day 0 and dropped to less than 0.5 by Day 6, albeit with wide 95% confidence intervals.

## 4. Discussion

### 4.1. Coverboard Surveys

During coverboard surveys, snake abundance varied both temporally and spatially. Day of year had a significant influence on seasonal abundance, with a peak during late May. *Clonophis kirtlandii* have been documented to mate primarily during spring [36,38,64] and this is correlated with seasonally high water tables that are an important habitat component for the species [42]. During this time, they are more mobile while searching for mates, which would increase their potential for detection in surveys. This time period aligned well with the peak of snake activity observed in our surveys and we observed multiple snakes under a single coverboard presumably involved in courtship behavior during this time.

Snake activity was also different between years, with far more snakes captured in 2017. This variation may also be tied to water level variation. Water levels were higher in 2017, thus closer to the coverboard transects. As with many natricine snakes, *C. kirtlandii* are often associated with close proximity to water [40]. Dry years or during later summer when water levels are usually low, *C. kirtlandii* are likely to follow the receding water or spend more time in refugia [36]. In this case, seasonal and annual low water levels likely caused snakes to move away from the stationary coverboard transects. 

When analyzing habitat variables, close shrub proximity and the presence of nearby grass and herbaceous vegetation were associated with snake presence at any given coverboard. *Clonophis kirtlandii* are generally defined as a wet meadow or marshland species [35,36]. These habitats are dominated by grasses and herbaceous vegetation, with interspersed shrubs. The *C. kirtlandii* at MNWR seem to follow this general habitat choice. Analysis of survey variables showed that coverboard transect group varied in snake captures, with the south group having the greatest chance of encountering a snake. While the exact driver for this was not found in the analyses conducted, the habitat variables that influence the distribution of *C. kirtlandii* are most likely associated with the macrohabitat variation that we did not characterize. Satellite imagery of MNWR reveals a large open wetland that encompasses the south transect group that is lacking at the middle and north transect groups. As *C. kirtlandii* are so poorly understood, MNWR would provide a valuable field location to further study macrohabitat choice in this species.

### 4.2. Environmental DNA Surveys

While difficult to find, 154 *C. kirtlandii* were captured in 42 coverboard surveys across the two seasons at MNWR. In comparison, we found at most six eDNA detections out of 380 environmental samples in four sampling events. At first glance, this suggests that eDNA does not offer an advantage over traditional survey methods in this system. However, we caution this outcome be considered tentative given methodological issues in assay performance and environmental sampling. 

The assay we developed fell short of expected standards of qPCR assays [65]. Efficiency was below 90% [66], the R^2^ of the standard curve was relatively low [65], and the relatively high LOD suggested that the assay lacked the sensitivity achieved in other eDNA studies [13,67]. There was also some suggestion of cross-amplification with non-target species, which may have further contributed to reduced sensitivity to low copy number eDNA. These assay characteristics occurred after a thorough effort to optimize qPCR conditions by varying annealing temperatures and primer and probe concentrations. Despite these limitations, we did find that the assay was sufficiently sensitive to detect Kirtland’s Snake eDNA under the semi-natural conditions of the degradation experiment (Table 3 and Figure 3) and a handful of field samples. Nonetheless, future work on the species will benefit from a redesign of primers and probes using a more complete characterization of mitochondrial sequence variation in natricines than what is currently available in public databases. This should result in increased sensitivity of the assay and greater confidence in the detection of Kirtland’s Snake DNA in environmental samples collected from the field.

Beyond assay characteristics, the method of environmental sampling influenced overall eDNA detection. Due to the uncertainty of snake eDNA distribution in semi-aquatic environments, we originally collected multiple sample types from fewer locations to explore the detectability of *C. kirtlandii* eDNA across several microhabitats. This imposed a tradeoff in the total number of samples able to be collected, thus limiting the number of locations that were sampled. This may have been detrimental to overall detection, as it became evident that sediment from crayfish burrows provided the best chances of eDNA detection. This is supported by samples collected from artificial burrows used in the degradation analysis (Table 3), and the few putative positive detections from natural crayfish burrows. Meanwhile, there were possibly two detections in open water and none in soil samples from burrow lips and under coverboards. Although soil samples did demonstrate qPCR inhibition (30% overall), a majority of samples were not inhibited and there were no detections in this sample type. Environmental DNA has been shown to persist far longer in sediment compared to that suspended in the water column [30,31] and our method of centrifuging the burrow water mixture prior to extraction likely led to the concentration of any eDNA or shed cells in the sample to the sediment pellet. Increasing the volume of water and sediment collected (>50 mL) may increase detection [68] from burrows, but this will likely require collection of groundwater adjacent to burrows since water levels were too low for collection in some burrows during drier periods. We used this information during the 2018 season by only collecting samples from crayfish burrows. Future eDNA studies on comparable species should similarly focus their efforts on collecting as many samples as possible from the most productive sample types, such as crayfish burrows and large volumes of open water, to have the greatest chance of species detection.

The degradation of eDNA in nature will also impact detection success. Our degradation experiment suggested that substantial degradation of *C. kirtlandii* eDNA occurs within a week, although it may persist for up to 31 days in crayfish burrow sediment (Table 3). These results are similar to that of other eDNA degradation studies in snakes performed under controlled laboratory conditions. Burmese Python (*Python bivvitatus*) eDNA began to degrade two days after snake removal and was 60% degraded after seven days [8]. Similarly, Red Cornsnake (*Pantherophis guttata*) eDNA has been shown to degrade in soil within a week [69]. This suggests that the temporal window for eDNA detection is brief and likely associated with recent snake activity.

Finally, although there are few eDNA studies on reptiles, it appears that detection rates are low relative to other aquatic vertebrates [22]. For example, two recent studies on Eastern Massasauga (*Sistrurus catenatus*), a species with an overlapping geographic range and similar use of wetlands and crayfish burrows [42,70], found limited eDNA detection. In a massasauga population in Illinois, two of 100 crayfish burrows were positive for eDNA [13] while a study in Michigan found one positive detection out of 60 paired crayfish burrow water and sediment samples [54]. The one snake species where eDNA detection has had some success is the Burmese Python [8,67,69], a species that is significantly larger in size than *C. kirtlandii* or *S. catenatus,* suggesting that eDNA detection may be a function of body size within snakes [71]. More broadly, the greater amount and frequency of shed skin, mucus, and waste from amphibians and fish are hypothesized to make their detection much more tractable relative to snakes and turtles in freshwater systems [22]. Research aimed at identifying where, when, and how snakes slough tissues into the environment will aid in the development of more targeted sampling schemes. For example, we assumed that feces would be a likely source of eDNA in Kirtland’s Snake in crayfish burrows but knowledge of whether snakes routinely defecate in burrows is lacking. Thus, the higher success of eDNA detection in the degradation experiment where artificial burrows were spiked with feces, compared to that of natural crayfish burrows, could be explained by snakes not shedding this and other biomaterials in crayfish burrows.

## 5. Conclusions

While eDNA sampling offers advantages over standard surveys in some species, detection in *C. kirtlandii* remains both technically and biologically challenging. More sensitive assays and greater volumes of sampled ground water are needed to boost confidence in the assessment of eDNA as a survey tool. Moreover, the possible low rate of tissue shedding in snakes and the rapid degradation of eDNA will require a very targeted approach to environmental sampling for successful detection. Our coverboard surveys show that sample collection early in the activity season in habitats with high water tables and predominantly grassy vegetation will aid in such an effort. Moreover, there is an acute need to better understand the behavior and activity of *C. kirtlandii* to increase the effectiveness of both eDNA and traditional surveys. 

## Figures and Tables

**Table 1:** Results of generalized estimating equation analysis using a negative binomial model to test the effect of survey variables on snake encounters. Transect group south and year 2018 were set to zero due to their inclusion in the intercept of the model.

Variable	β	95% Confidence Interval		Wald χ^2^	df	*p*
		Lower	Upper			
Intercept	0.921	−0.913	2.755	0.970	1	=0.325
North transects	−3.241	−4.623	−1.859	21.117	1	<0.0001
Middle transects	−2.714	−4.152	−1.277	12.694	1	<0.0001
South transects	0					
Year 2017	1.977	1.038	2.917	17.015	1	<0.0001
Year 2018	0					
Day of year	−0.020	−0.030	−0.009	14.224	1	<0.0001
Survey start time	0.123	−0.021	0.267	2.793	1	=0.095
Cloud cover (%)	0.002	−0.002	0.006	0.890	1	=0.345
Air temp (°C)	−0.013	−0.044	0.018	0.658	1	=0.417

**Table 2:** Binary logistic regression of habitat variables related to snake presence. Percent ground cover water, rock, and bare ground were omitted from the analysis due to high zero counts.

Variable	β	SE	Wald χ^2^	df	*p*=
Constant	−29.120	15.264	3.640	1	0.056
Shrub distance (m)	−0.531	0.259	4.187	1	0.041
Log distance (m)	−0.094	0.051	3.415	1	0.065
Grass (%)	0.316	0.157	4.050	1	0.044
Herbaceous (%)	0.309	0.156	3.919	1	0.048
Shrub (%)	0.298	0.155	3.684	1	0.055

**Table 3:** *Clonophis kirtlandii* detections from artificial crayfish burrows (n = 10) in degradation study. Average (± standard deviation) values of water volume, temperature, dissolved oxygen, and pH are given for each sample day. The number of detections for burrow water and burrow sediment are given for each of two thresholds for assigning a positive (two of three and one of three qPCR replicates). Samples were unable to be collected at 17 days.

Day of Sample	Water Volume (mL)	Temperature (^◦^C)	Dissolved Oxygen (%)	pH	Burrow Water Detections (2 of 3)	Burrow Water Detections (1 of 3)	Burrow Sediment Detections (2 of 3)	Burrow Sediment Detections (1 of 3)
Pre-spike	523.4 (±60.5)	14.1 (±0.7)	37.4 (±7.6)	5.9 (±0.2)	0	0	0	0
Post-spike	473.4 (±60.5)	14.1 (±0.7)	37.4 (±7.6)	5.9 (±0.2)	1	2	7	7
1	355.7 (±98.9)	18.3 (±2.7)	22.3 (±6.7)	6.1 (±0.3)	0	0	8	9
2	296.1 (±80.1)	18.5 (±1.9)	22.1 (±6.1)	6.2 (±0.3)	0	0	7	7
3	225.9 (±65.4)	24.8 (±1.6)	14.7 (±5.4)	6.2 (±0.2)	0	0	7	7
10	181.7 (±64.8)	22.1 (±1.2)	16.4 (±3.2)	6.3 (±0.2)	0	0	2	3
17	-	-	-	-	-		-	-
25	430.3 (±55.6)	20.1 (±0.7)	22.2 (±5.8)	5.7 (±0.3)	0	0	1	2
31	251.9 (±85.9)	30.2 (±1.1)	15.7 (±7.8)	6.2 (±0.2)	0	1	0	2

## Supplementary Materials

The following are available online at https://www.mdpi.com/2076-2615/10/6/1057/s1, Figure S1: Quantitative PCR amplification of 0.1 ng/µL concentration tissue extracted snake DNA, Figure S2: Quantitative PCR amplification of *C. kirtlandii* tissue and feces positives, and eDNA from a crayfish burrow, Figure S3: Cumulative standard curve from all qPCR standards, Table S1: Comparison of primer and probe sequences of the eDNA assay to homologous CO1 sequences acquired from GenBank. Table S2: Correlation matrix of potential predictors of eDNA detection in artificial crayfish burrows.
