# Supplementary material for: Exploration of Environmental DNA (eDNA) to Detect Kirtland’s Snake (Clonophis kirtlandii)
Source: Animals (Basel). 2020 Jun 19;10(6):1057. doi: 10.3390/ani10061057 (PMC7341209; doi:10.3390/ani10061057)

**Supplemental Figure 1**. Quantitative PCR amplification of 0.1 ng/µL concentration tissue extracted snake DNA. *Clonophis kirtlandii* DNA amplification occurred well before that of the non-target species *S. dekayi*, *T. sirtalis*, and *T. sauritus*.


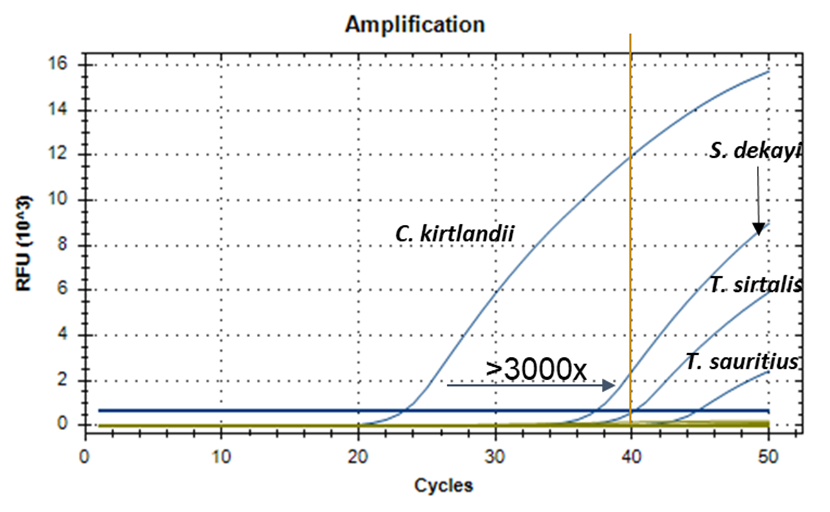

Supplement: Supplementary file 1 [file animals-10-01057-s001.zip › Figure S1.docx]
