# Supplementary material for: Exploration of Environmental DNA (eDNA) to Detect Kirtland’s Snake (Clonophis kirtlandii)
Source: Animals (Basel). 2020 Jun 19;10(6):1057. doi: 10.3390/ani10061057 (PMC7341209; doi:10.3390/ani10061057)
Supplement: Supplementary file 1 [file animals-10-01057-s001.zip › Figure S2.docx]

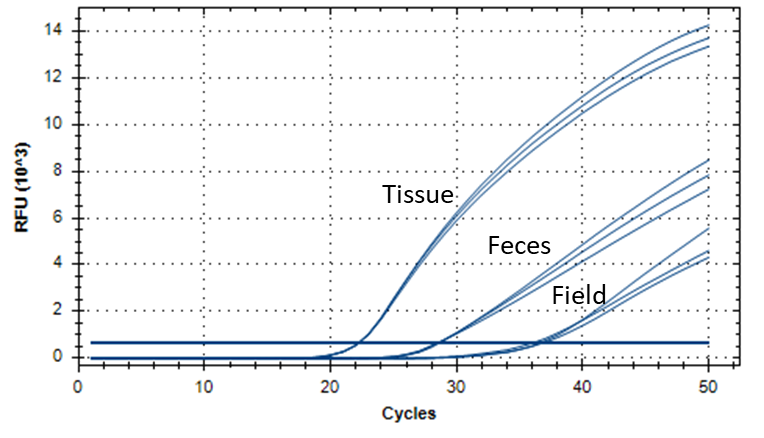


**Supplemental Figure 2**. Quantitative PCR amplification of *C. kirtlandii* tissue and feces positives as well as a positive detection of *C. kirtlandii* eDNA from crayfish burrow sediment in spring 2017.
