# Supplementary material for: Exploration of Environmental DNA (eDNA) to Detect Kirtland’s Snake (Clonophis kirtlandii)
Source: Animals (Basel). 2020 Jun 19;10(6):1057. doi: 10.3390/ani10061057 (PMC7341209; doi:10.3390/ani10061057)
Supplement: Supplementary file 1 [file animals-10-01057-s001.zip › Figure S3.docx]

**Supplemental Figure 3**. Cumulative standard curve from all qPCR standards. No detections were recorded at or below 1x10^1^ copy number standards.

**
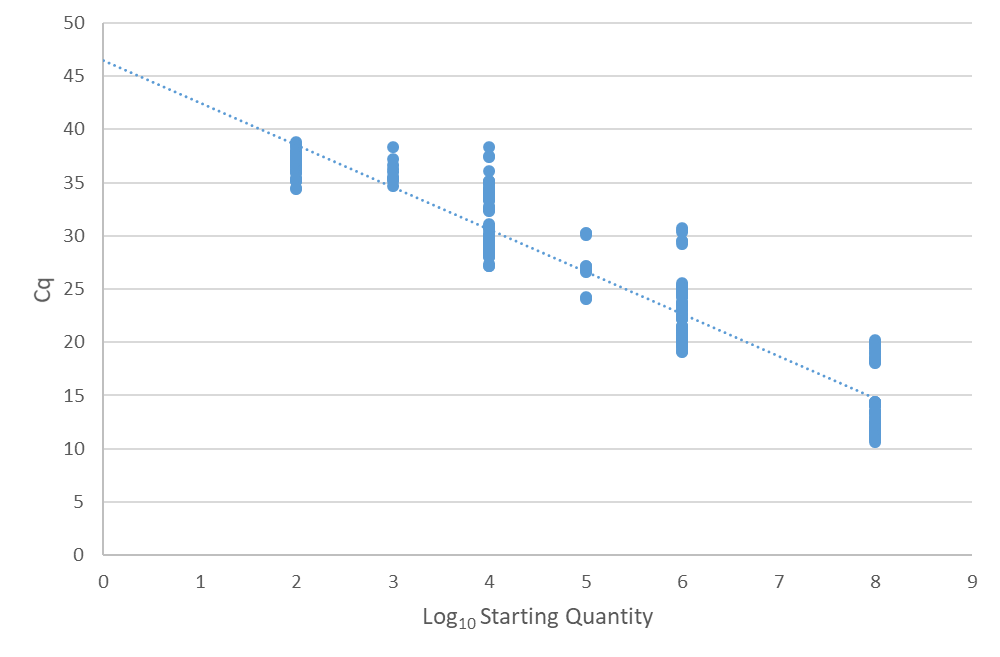
**
