# Supplementary material for: Exploration of Environmental DNA (eDNA) to Detect Kirtland’s Snake (Clonophis kirtlandii)
Source: Animals (Basel). 2020 Jun 19;10(6):1057. doi: 10.3390/ani10061057 (PMC7341209; doi:10.3390/ani10061057)
Supplement: Supplementary file 1 [file animals-10-01057-s001.zip › Table S2.docx]

**Supplemental Table 2**. Correlation matrix of potential predictors of eDNA detection in artificial crayfish burrows (*p< 0.05, **p<0.01).
